# Evaluating BMI, Upper Airway Dimensions, and Hyoid Bone Position and their Correlation in Non-OSA Snoring Adults: the First CBCT Study 

**DOI:** 10.30476/dentjods.2024.102409.2360

**Published:** 2025-06-01

**Authors:** Sogol Jafari-Pozve, Nasim Jafari-Pozve, Ali Farzamfar, S. Marjan Arianezhad, Seyed Sasan Aryanezhad

**Affiliations:** 1 Dentist, School of Dentistry, Isfahan (Khorasgan) Branch, Islamic Azad University, Isfahan, Iran.; 2 Dept. of Oral and Maxillofacial Radiology, School of Dentistry, Isfahan (Khorasgan) Branch, Islamic Azad University, Isfahan, Iran.; 3 Postgraduate Student Dept. of Oral and Maxillofacial Radiology, Student Research Committee, School of Dentistry, Shahid Beheshti University of Medical Sciences, Tehran, Iran.; 4 Dept. of Oral and Maxillofacial Radiology, School of Dentistry, Islamic Azad University of Isfahan (Khorasgan), Isfahan, Iran.

**Keywords:** Snoring, Obstructive sleep apnea, Body Mass Index, Hyoid Bone, Cone-Beam Computed Tomography

## Abstract

**Background:**

Precise knowledge about the characteristics of individuals who snore but do not have obstructive sleep apnea (OSA) is essential yet remains limited in the literature.

**Purpose:**

This study aimed to evaluate BMI (body mass index), upper airway dimensions, hyoid bone position, and their relationship in non-OSA snoring adults using cone-beam computed tomography.

**Materials and Method:**

In this retrospective cross-sectional study, cone beam computed tomography (CBCT) records of 100 patients were analyzed. Patients were snoring non-OSA individuals, diagnosed through a thorough examination and negative polysomnography results. CBCT scans were executed in the standard position as per the specified protocol of the Sleep Center. Upper airway was divided into four parts (nasopharynx, velopharynx, oropharynx, and hypopharynx), and anteroposterior (AP) and transverse (T) dimensions within the minimal cross-sectional area of each respective region were evaluated. BMI, upper airway dimension, and hyoid bone were analyzed using Pearson and Spearman's correlation tests.

**Results:**

The retrognathion-hyoid-4th cervical vertebra (RHV) angle representative of the hyoid position was significantly greater in females (*p* Value=0.000). It also statistically decreased significantly in snorers aged 36-50 and 51-65, *p*= 0.006 and 0.012, respectively. Snorers had above-average BMI in all age groups and both genders. The AP-hypopharynx significantly correlated with BMI (*p*= 0.022).

**Conclusion:**

The hyoid position was gender-dependent, with a superior position in females. It was also age-dependent, with a more inferior position in snorers aged 36-65 compared to younger ages (20-35). The AP-velopharynx is a constriction region in the airway of snorers. Snorers were overweight in all age groups and both genders. BMI had a significant correlation with the AP-hypopharynx.

## Introduction

Snoring is a common complaint among adults in the general population and can be a primary symptom of obstructive sleep apnea (OSA) [ 1 ]. Snoring is a growing healthcare concern due to its negative impact on the quality of life for both snorers and their family members [ 2 - 3 ]. OSA syndrome is caused by periodic obstruction of the upper airways, particularly the oropharynx, and is associated with transient nocturnal desaturation. Common signs and symptoms of OSA include loud snoring, sleep disturbances, diminished sleep quality, fatigue, and excessive daytime sleepiness. OSA syndrome often goes unnoticed for a long time because respiratory disorders occur at night, yet their consequences impact daily functioning [ 4 ]. OSA affects 4% of males and 2% of females. It has been demonstrated that obesity and cranial and facial morphology are critical factors in susceptibility to OSA [ 5 - 6 ]. 

In the past three decades, obesity has increased by 27.5% in adults and 47.1% in children [ 7 ]. The body mass index (BMI) values≥ 30 indicate obesity [ 7 ]. Obesity is the most significant risk factor for OSA, but it is also reversible. OSA and obesity significantly correlate and can predispose patients to other diseases [ 8 ]. 

The pathogenesis of snoring is closely related to the anatomy of the upper airway. The hyoid bone is located behind the jaw and in front of the cervical vertebrae. Changes in the anteroposterior position of the head and mandibular slope can affect the hyoid bone position. It has been demonstrated that inferior positioning of the hyoid bone (compared with the mandibular plane) significantly correlates with OSA. In addition, the hyoid bone is a commonly used landmark for measuring the pharyngeal height and upper airway dimensions . 

Cone-beam computed tomography (CBCT) is a cost-effective and easily accessible method for three-dimensional jaw evaluation and quantitative measurements [ 12 - 13 ]. Since the airway is a region of no attenuation, its boundaries are accurately detected in CBCT. It has been widely favored due to its shorter acquisition time compared to CT scans, reducing the chance of patient movement such as during breathing, swallowing, or other involuntary movements [ 14 - 16 ]. Most previous studies have been conducted on CBCT scans taken in the supine position since the sleep apneic events occur in the supine position during sleep [ 17 ]. However, it has been suggested that CBCT imaging of supine patients is inappropriate as it fails to replicate conditions during sleep [ 17 - 18 ]. Dentists, who frequently see their patients more often than physicians, are well-positioned to be the first to identify potential sleep issues. Moreover, the common use of CBCT in dentistry further empowers dentists to assess these conditions effectively. Therefore, dentists need to have a thorough understanding of sleep disorders and the skills to evaluate their patients; ensuring timely referrals or appropriate treatments are provided [ 32 ]. Considering the numerous problems that snoring causes in terms of quality of life and its potential to develop into OSA, it is important to conduct research on individuals who snore. OSA as a prevalent sleep-breathing disorder, affects an estimated 1 billion people of all ages globally [ 8 ]. It is closely associated with several serious illnesses, including arterial hypertension, cardiovascular disease, stroke, and metabolic syndrome [ 19 - 20 ]. On the other hand, the exact mechanism by which OSA develops is not completely understood. De-spite extensive research on OSA patients [ 17 ], there is a notable lack of literature evaluating the characteristics of individuals who snore but do not have OSA. To the authors’ best knowledge, it is the first study to evaluate the airway configurations in non-OSA snoring patients and explore the relationship between body BMI and the airway structure and the positioning of the hyoid bone. 

## Materials and Method

This study was a retrospective cross-sectional study that followed the guidelines of the Declaration of Helsinki and its subsequent revisions. The study protocol was approved by the University's Ethics Committee under the code number IR.IAU.KHUISF.REC.1401.151. Additionally, the study was conducted in accordance with the STROBE statement.

CBCT images of patients who attended a sleep laboratory center at Iran University of Medical Sciences in Tehran between 2019 and 2022 were retrieved and assessed. All the patients were snoring non-OSA individuals, as diagnosed through a thorough examination by the physicians, followed by CBCT imaging and negative polysomnography results. None of the patients received additional X-rays, and the physician prescribed the request for CBCT to assess the paranasal sinuses. The institution’s policy includes patients’ consent to participate in any trial and approvals of using their data with full clarification of the benefits and risks of any procedure.

The minimum sample size was calculated to be 100 according to a previous study by Tseng *et al*. [ 10 ], assuming α=0.05, β=0.2, and a study power of 80%. As the study was conducted retrospectively, selection bias was a potential issue. To reduce the impact of this bias, 900 CBCT scans were initially evaluated for eligibility, and only 100 of them were chosen based on the predefined eligibility criteria. In addition, demographic data such as age and gender were documented for each selected case.

### Inclusion criteria [ 21 ] 

Confirmed snoring non-OSA patients by physician, negative polysomnography result, document of CBCT of the patient with adequate FOV, age of 20-65, and image with adequate quality without artifacts were considered s inclusion criteria.

### Exclusion criteria [ 22 ]

Patients with a history of asthma, COPD, history of cerebrovascular disease, symptomatic ischemic, patients with a history of heart disease, congestive heart failure, history of chronic renal failure, history of hypothyroidism, rheumatologic diseases, with nasal, oral, pharyngeal, or mandibular diseases, patients who had been treated for OSA, evidence of previous maxillofacial surgery and trauma on CBCT scans, developmental and congenital anomalies, history of asthma or sinusitis (acute or chronic), diseases associated with airway inflammation such as the common cold and influenza, and pregnancy were considered as exclusion criteria.

All CBCT scans were obtained using a Galilieos-Sirona CBCT scanner (Sirona Dental Systems GmbH, Bensheim, Germany) in high-resolution mode, with the exposure settings of 85-100 kV, 5-7 mAs, total filtration of 2.5 mm Al, and 14 seconds of scanning time. 

All CBCT scans conducted for airway evaluation were executed in the standard position as per the specified protocol. The subject stood upright with their head in a natural position, aligned with the horizontal visual axis with maximal intercuspation of the teeth and lips in light contact and natural head position as the standardized position [ 23 ]. A cross-light beam projected onto the face prevented any possible lateral head tilt or rotation. Finally, the head holder stabilized the head posture during exposure. Therefore, postural variations in the upper airway dimension were eliminated, and accurate comparisons were made. Currently, most commercial dental CBCT units acquire images with the patient upright [ 18 ]. Hence, it is necessary to assess the airway morphology of individuals in a similar manner. Considering this, it was decided to assess the variables in an upright CBCT scanner.

The images were then assessed using SIDEXIS soft-ware version 4.1 (3D Viewer; Sirona, Germany) in an Intel Core i7-4460 at 3.20 GHz (Intel Corp, Santa Clara, CA) PC workstation running Windows 10 professional SP-2 (Microsoft Corp, Redmond, WA).

An expert oral and maxillofacial radiologist (S.M.A.) evaluated all the measurements in this study. However, the subjective nature of the measurements introduced some bias. To ensure the accuracy of the measurements, the intra-observer agreement was assessed using the intra-class coefficient (ICC) test. To perform this test, 20 CBCT scans were evaluated twice, with a 1-month interval between evaluations. Additionally, all stages of the image examination were conducted blindly to minimize any potential bias.

The CBCT scans of patients were divided into 20-35-, 36-50-, and 51-65-year-old age groups. 

The participants' BMI (kg/m2) was calculated by dividing their weight in kilograms as measured by a digital scale by the square of their height in meters. In accordance with the definition of snoring specified in the S-TOP-Bang questionnaire, we used the criterion: 'Do you snore loudly (louder than talking or loud enough to be heard through closed doors)?' This standardized definition guided our assessment of snoring in the study [ 24 ].

**Figure 1:**
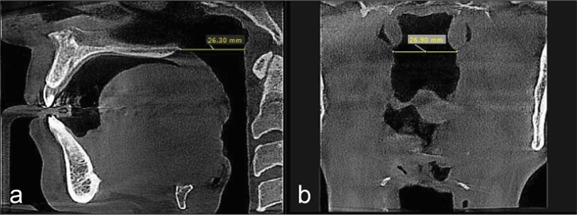
**a:** Measuring the minimum anteroposterior dimension of the nasopharynx in the sagittal plane; **b:** minimum transverse dimension of the nasopharynx in the coronal plane

**Figure 2:**
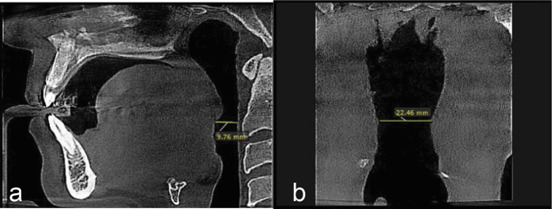
**a:** Measuring the minimum anteroposterior dimension of the velopharynx in the sagittal plane; **b:** Minimum transverse dimension of the velopharynx in the coronal plane

**Figure 3:**
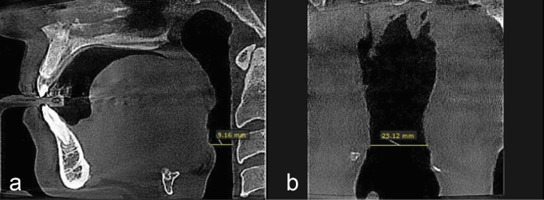
**a:** Measuring the minimum anteroposterior dimension of the oropharynx in the sagittal plane; **b:** Minimum transverse dimension of the oropharynx in the coronal plane

**Figure 4:**
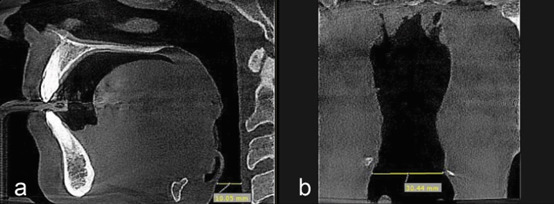
**a:** Measuring the minimum transverse dimension of the hypopharynx in the coronal plane; **b:** minimum anteroposterior dimension of the hypopharynx in the sagittal plane

**Figure 5:**
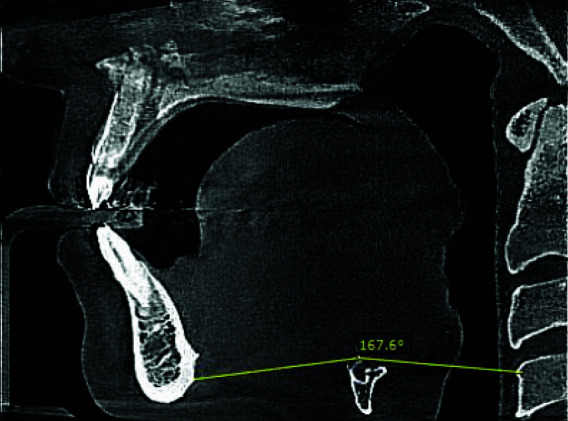
Position of hyoid triangle according to the RHV angle

The upper airway was segmented into four subregions, and the definition of each is detailed in Tables 1-2. The minimum cross-sectional area of each subregion was accurately identified by scrolling through axial, coronal, and sagittal sections, with the conclusive measurement taken from the axial section. The anteroposterior (AP) and transverse (T) dimension within the minimum cross-sectional area of each respective region was calculated, resulting in min-AP and min-T of each subregion (Figures 1-5). 

**Table 1:** Anatomical limits of Upper airway

Region	Limits	Anatomical	Technical
Nasopharynx	Anterior	Frontal plane perpendicular to FH passing through PNS	=
	Posterior	Soft tissue contour of the pharyngeal wall	Frontal plane perpendicular to FH passing through C2sp
	Upper	Soft tissue contour of the pharyngeal wall	Top of the upper airway: Plane parallel to FH passing through the last axial slice before the nasal septum fused with the posterior pharyngeal wall
	Lower	Plane parallel to FH passing through PNS and extended to the posterior wall of the pharynx	=
	Lateral	Soft tissue contour of the pharyngeal wall	Sagittal plane perpendicular to FH passing through the lateral walls of the maxillary sinus
Velopharynx	Anterior	Frontal plane perpendicular to FH passing through PNS	=
	Posterior	Soft tissue contour of the pharyngeal wall	Frontal plane perpendicular to FH passing through C2sp
	Upper	Lower limit of Nasopharynx	=
	Lower	Plane parallel to the FH plane passing through the tip of the uvula	=
	Lateral	Soft tissue contour of the pharyngeal wall	Sagittal plane perpendicular to FH passing through the lateral walls of the maxillary sinus
Oropharynx	Anterior	Frontal plane perpendicular to FH passing through PNS	=
	Posterior	Soft tissue contour of the pharyngeal wall	Frontal plane perpendicular to FH passing through C2sp
	Upper	Lower limit of Velopharynx	=
	Lower	Plane parallel to the FH plane passing through the tip of the epiglottis	=
	Lateral	Soft tissue contour of the pharyngeal wall	Sagittal plane perpendicular to FH passing through the lateral walls of the maxillary sinus
Hypopharynx	Anterior	Frontal plane perpendicular to FH passing through PNS	=
	Posterior	Soft tissue contour of the pharyngeal wall	Frontal plane perpendicular to FH passing through C2sp
	Upper	Lower limit of Oropharynx	=
	Lower	The plane connecting the entrance of the esophagus to the body of the hyoid bone and the left and right greater horns of the hyoid.	=
	Lateral	Soft tissue contour of the pharyngeal wall	Sagittal plane perpendicular to FH passing through the lateral walls of the maxillary sinus

**Table 2:** Definition of landmarks used in the study

Landmark	Definition
H point	The most prominent point of the superior-anterior border of the body of the hyoid bone
R point	The most posterior-inferior point of the mandibular symphysis (RGN)
V point	The most anterior-superior point of the 4th cervical vertebra (VC4)

The normal distribution of data was evaluated by the Shapiro-Wilk test. Pearson and Spearman's correlation coefficients were used to analyze the relationship of BMI with normally and non-normally distributed upper airway dimensions, respectively. An independent t-test was utilized to compare the position of the hyoid bone in males and females. Additionally, a two-way ANOVA was conducted to analyze the effect of BMI on the position of the hyoid bone in males, females, and different age groups. All statistical analyses were performed utilizing SPSS version 26 at a 0.05 significance level. 

## Results

Measurements for the first and second replicates of 20 patients were recorded, and the ICC values were established for all measurements. Most measures showed high reliability between the first and second replicates, with ICC values ranging from 0.81 to 0.98. 

**Figure 6:**
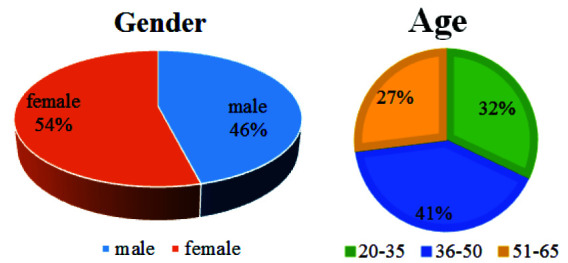
Distribution of gender and age

In this study, 100 patients (54 women and 46 men) underwent CBCT imaging, as shown in Figure 6. The subjects' ages ranged from 20 to 65 years; accordingly, they were divided into three groups: 20-35, 36-50, and 51-65.

**Figure 7:**
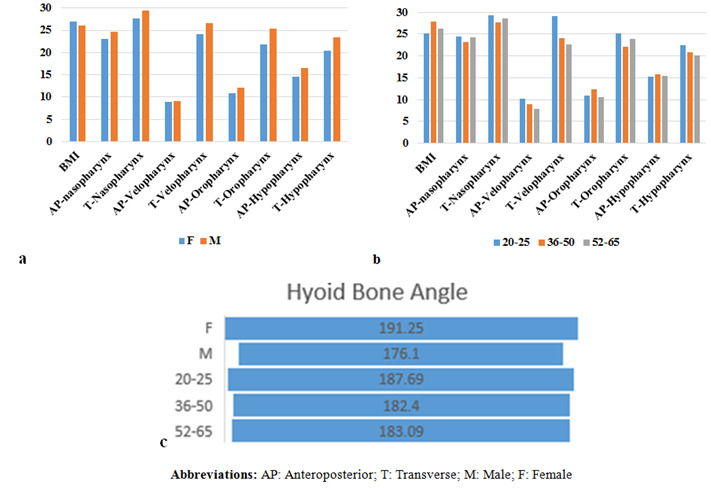
**a:** Prevalence of BMI and upper airway dimensions by gender, **b:** Age group and c: Hyoid bone by gender and age

Figure 7 provides a summary of the prevalence of BMI, upper airway dimensions, and hyoid bone position in our study. The min-AP dimension of the velopharynx was identified as the most constricted area of the four subregions of the airway in both males and females. When considering age groups, the min-AP of velopharynx was found to be the most constricted in individuals aged 36-50. As depicted, the participants exhibited above-average BMIs in all age groups and both genders, suggesting that snoring non-OSA adults were, on average, overweight. The results revealed that BMI had a significant correlation with the min-AP of the hypopharynx (r=-0.229, *p*= 0.022), indicating that as BMI increased, AP dimension of the hypopharynx decreased. 

However, BMI had no statistically significant associations with the min-AP of the nasopharynx (r= -0.059, *p*= 0.558), the min-T of the nasopharynx (r=-0.059, *p*= 0.558), the min-AP of the velopharynx (r=-0.052, *p*= 0.609), the min-T of the velopharynx (r=-0.192, *p*= 0.056), the min-AP of the oropharynx (r= 0.063, *p*= 0.532), the min-T of the oropharynx (r=-0.103, *p*= 0.307), the min-T of the hypopharynx (r=--0.103, *p*= 0.084), and the hyoid bone position (r= 0.102, *p*= 0.312). Independent t-test showed a significant difference in hyoid bone position between males and females (*p*= 0.000), concluding that the retrognathion-hyoid-4th cervical vertebra (RHV) angle (Table 2-3) is significantly greater in females than males, meaning that females had superior position of hyoid compared to males (Figure 7). The hyoid bone was also age-dependent meaning that with an increase in age, the hyoid bone angle changed, and a significant difference was noted in the mean size of RHV angle in 36-50 and 51-65-year-old individuals, *p*= 0.006 and 0.012, respectively, meaning that the hyoid position was more inferiorly in this age group. Two-way ANOVA showed no significant effect of gender (*p*= 0.409), hyoid bone angle (*p*= 0.970), or their interaction (*p*= 0.971) on BMI. Table 4 presents the correlation coefficients for the relationship between BMI and upper airway dimensions by gender. As indicated, BMI had no significant correlation with upper airway dimensions in males and females and the correlations were not significantly different between males and females (*p*> 0.05). 

**Table 3:** Prevalence of BMI, upper airway dimensions, and hyoid bone position (n=100) in our study

Variable	Minimum	Maximum	Mean± Std. deviation
BMI (kg/m2)	16.30	35.83	26.55±4.146
AP- nasopharynx (mm)	13.32	32.21	23.85±3.364
T- nasopharynx (mm)	14.62	37.77	28.44±4.504
AP- velopharynx (mm)	4.13	15.66	9.07±2.632
T- velopharynx (mm)	10.69	43.11	25.31±6.694
AP- oropharynx (mm)	3.92	30.52	11.46±3.850
T- oropharynx (mm)	12.12	41.33	23.47±6.365
AP- hypopharynx (mm)	4.18	20.08	12.13±1.210
T- hypopharynx (mm)	11.66	33.11	22.38±3.806
Linear angle of hyoid bone (degrees)	134.50	223.60	184.28±18.313

Abbreviations: AP, Anteroposterior; T, transverse

**Table 4:** Correlation coefficients for the correlation of BMI with upper airway dimensions by Gender

Variable	Gender	Pearson’s correlation coefficient	Spearman’s correlation coefficient	*p* Value
AP-nasopharynx	F	0.088	-	0.526
	M	-0.135	-	0.370
T-nasopharynx	F	-	-0.033	0.813
	M	-0.126	-	0.403
AP-velopharynx	F	-0.160	-	0.247
	M	0.053	-	0.727
T-velopharynx	F	-0.052	-	0.708
	M	-0.149	-	0.324
AP-oropharynx	F	0.097	-	0.486
	M	-	0.167	0.267
T-oropharynx	F	0.086	-	0.537
	M	-0.226	-	0.131
AP-hypopharynx	F	0.256	-	0.053
	M	0.275	-	0.065
T-hypopharynx	F	0.058	-	0.679
	M	-0.337	-	0.022

Abbreviations: AP, Anteroposterior; T: Transverse; M: Male; F: Female

BMI had no significant correlation with hyoid bone position (angle) in 30-40 (r=-0.194, *p*= 0.287), 41-51 (r= 0.213, *p*= 0.181), and 52-62 (r= -0.96, *p*= 0.635) year-olds. Two-way ANOVA showed no significant effect of age group (*p*= 0.058), hyoid bone angle (*p*= 0.635), or their interaction (*p*= 0.411) on BMI. Table 5 presents the correlation coefficients between BMI and upper airway dimensions across different age groups. As indicated, BMI had no significant correlation with upper airway dimensions in the three age groups and the correlations were not significantly different among the three age groups (*p*> 0.05). 

**Table 5:** Correlation coefficients for the correlation of BMI with upper airway dimensions by age groups

Variable	Age group (yrs)	Pearson’s correlation coefficient	Spearman’s correlation coefficient	*p* Value
AP- nasopharynx	20-35	0.043	-	0.817
	36-50	-0.071	-	0.658
	51-65	0.018	-	0.927
T-nasopharynx	20-35	-	0.310	0.084
	36-50	-0.238	-	0.133
	51-65	-0.056	-	0.782
AP- velopharynx	20-35	-0.155	-	0.389
	36-50	-	-0.168	0.294
	51-65	0.257		0.195
T-velopharynx	20-35	0.029	-	0.875
	36-50	-0.277	-	0.080
	50-65	-0.069	-	0.734
AP-oropharynx	20-35	-0.066	-	0.721
	36-50	-	-0.013	0.935
	51-65	0.214	-	0.283
T-oropharynx	20-35	0.225	-	0.216
	36-50	-0.264	-	0.095
	51-65	-0.069	-	0.732
AP-hypopharynx	20-35	0.093	-	0.612
	36-50	0.196	-	0.219
	51-65	0.349	-	0.074
T-hypopharynx	20-35	-0.091	-	0.619
	36-50	-0.178	-	0.267
	51-65	-0.089	-	0.659

Abbreviations: AP, Anteroposterior; T, transverse;

## Discussion

Snoring is a significant healthcare problem, causing dis-disturbances for many people worldwide because of its detrimental effects on the quality of life for both those who snore and their family members. Moreover, it is a primary symptom of OSA and can progress into the disorder itself. OSA is a prevalent disorder with potentially life-threatening consequences if left undiagnosed [ 1 - 4 ]. We aimed to evaluate BMI, the upper airway configurations, and the hyoid bone position of non-OSA snoring patients, utilizing CBCT scans to gain precise knowledge about their anatomical properties. Additionally, we investigated the relationship between BMI and these configurations.

The individuals included in this study demonstrated above-average BMIs in all age groups, suggesting they were overweight. Previous studies [ 25 - 27 ] have established a correlation between OSA and elevated BMIs. To the authors’ best knowledge, no work has been performed regarding the relationship of BMI and non-OSA snorers. Recent researches [ 28 ] indicate that the relationship between snoring and obesity has not been well explored. Our findings indicate that non-OSA snoring adults tend to have above-average BMIs in all age groups. Considering these results, we suppose that BMI assessment could potentially serve as one of the factors for determining the risk of snoring. By identifying individuals with higher BMI who are at risk, targeted weight loss could be planned for them as one of the programs to mitigate their snoring. This proactive approach may not only improve the quality of life for snorers but also benefit their family members by reducing the disturbances associated with snoring. This finding was consistent with the former study [ 28 ] that found a significant relationship between snoring and obesity. Additional research is suggested to thoroughly comprehend the predictive factors underlying OSA and snoring conditions and their relationship with BMI.

Our result showed that the AP-velopharynx is a region of constriction in the airway of snorers. It can be suggested that when evaluating the airway using CBCT, dentists and oral and maxillofacial radiologists should pay particular attention to the velopharynx. This area should be meticulously assessed. In cases where intervention for snoring is being considered, it is crucial to thoroughly evaluate this region and inform the surgeon of any potential constriction. We also found a signifi cant correlation between the AP- hypopharynx and BMI. In addition, the hyoid bone position was gender-dependent and had a superior position in females but no significant correlation with BMI was observed. To the authors’ best knowledge, this is the first study intended to evaluate the upper airway dimensions and hyoid bone position in non-OSA snorers using CBCT. The results of previous studies regarding the correlation of BMI with upper airway dimensions and hyoid bone position have been controversial. Junior *et al*. [ 11 ] reported that obese individuals had larger soft palate dimensions. In addition, the hyoid bone had a more posterior position in thin individuals. Korkmaz *et al*. [ 19 ] found no significant correlation between BMI with hyoid bone position and pharyngeal airway dimensions. Thapa *et al*. [ 30 ] reported that craniofacial parameters such as the hyoid bone position, longer tongue, and increased soft tissue thickness were correlated with narrowing of the upper airway in the oropharynx and hypopharynx, which may be responsible for OSA. Sutherland *et al*. [ 31 ] reported that changes in the length of the upper airways have a greater impact on reducing the frequency of apnea attacks. Our result is in line with this study. In conclusion, the relationship between BMI and the min-AP of the hypopharynx indicates that this part of the airway is the most susceptible to changes associated with weight.

A review by Ahmed Masoud *et al*. [ 32 ] indicates that limited two-dimensional and three-dimensional norms exist for adult patients with sleep-disordered breathing (SDB), and even fewer exist for children. It highlights a lack of research in the field of SDB and the mechanisms through which it develops. Since CBCT can meticulously show the boundaries of the upper airway because the airway has no attenuation in CBCT, we recommend conducting more research using this advanced imaging technique on snorers and OSA patients.

Most previous studies have emphasized the significant effect of hyoid bone position on upper airway dimensions [ 33 - 36 ]. In the present study, as age increased, the hyoid bone angle changed significantly. Notably, the mean size of the RHV angle decreased statistically significantly in the 36-65 age group compared to the 20-35 age group, meaning that it had a more inferior position in older snorer groups. Yu *et al*. [ 33 ] demonstrated the more inferior position of the hyoid bone can aggravate OSA. It is also found that males are more susceptible to OSA than females [ 36 ]. Alongside the results of our study, there appears to be a relationship between hyoid position and susceptibility to snoring. Our findings align with previous studies [ 33 - 34 ] indicating that older and male snorers tend to have a more inferior hyoid position. Further investigation is warranted to elucidate the role of the hyoid bone as a predisposing factor for snoring and the development of OSA.

Two notable challenges confronted our study. Firstly, defining the exact boundaries of the upper airway subregion proved challenging due to variations among different studies. To address this, subregion definitions with bony landmarks were adopted, with technical feasibility and statistical reliability, especially for three-dimensional calculations, which were employed in our study. Secondly, another challenge pertained to head positioning. The airway in CBCT is position-dependent, meaning that the mandible's and tongue's positioning can significantly influence the dimensions of the upper airway, especially the oropharynx. Various head positions have been employed in previous studies. In our efforts to minimize the impact of head position on airway dimensions, we opted for a standardized head position used in the Sleep Center based on protocols established by Solow *et al*. [ 23 ] and Guijarro-Martínez *et al*. [ 17 ]. Moreover, by employing a CBCT scan in an upright position, a cephalostat was used to stabilize the head and eliminate postural variations in the upper airway dimensions, allowing for accurate comparisons. 

### Limitations and suggestions

This study has some limitations. First, since it was a retrospective study, it was difficult to clearly classify complex clinical symptoms. Second, there was a lack of similar studies specifically focusing on the anatomical properties of snorers, which limited the availability of comparative data. As a result, future prospective studies, longitudinal research, and larger sample sizes in the field of snoring and OSA patients are recommended to investigate the exact mechanisms and their relationships. Furthermore, evaluation of soft tissues, including the tongue and uvula, along with other anatomical angular and linear dimensions such as SNA and SNB, is suggested to comprehensively assess the underlying factors contributing to adult snoring.

## Conclusion

The AP-velopharynx is a constriction region in the air way of snorers. The hyoid bone position was gender-dependent and had a superior position in females. It had a more inferior position in subjects aged 36-65 compared to the younger age group (20-35). As BMI increased, the AP-hypopharynx decreased. Snoring adults had above-average BMI in all age groups and both genders, suggesting that they were overweight.
